# Behavioral investigation of reactive and neoplastic lymphocytes in human lymph nodes in 4D

**DOI:** 10.1371/journal.pone.0331439

**Published:** 2025-09-04

**Authors:** Diana Elisa Theil, Cornelius Bütow, Sonja Scharf, Hendrik Schäfer, Sylvia Hartmann, Martin-Leo Hansmann, Patrick Wurzel

**Affiliations:** 1 Institute for general pharmacology and toxicology, Goethe University, University Hospital, Frankfurt/Main, Hesse, Germany; 2 Molecular bioinformatics, Goethe University, Frankfurt/Main, Hesse, Germany; 3 Dr. Senckenberg Institute of Pathology, Goethe University, University Hospital, Frankfurt/Main, Hesse, Germany; 4 Frankfurt Institute for Advanced Studies (FIAS), Frankfurt/Main, Hesse, Germany; Universita degli Studi di Roma La Sapienza, ITALY

## Abstract

This study deals with a 4D investigation of lymphocytes in human tissue under reactive and neoplastic conditions. The immune system’s response to pathogens highly depends on cell interaction and movement, which makes it essential to analyze these dynamics. To achieve this, we observed cells and their movement in 4D. Human lymphoid tissue was examined, including 8 cases with 23 movies of hyperplastic tissue from the pharyngeal tonsil and 12 cases with 35 movies of lymphadenitis. Additionally, there were 4 cases involving 16 movies of marginal zone lymphoma (MZL), 3 cases with 19 movies of nodular lymphocyte predominant Hodgkin lymphoma (NLPHL), 3 cases with 6 movies of follicular lymphoma grade 1/2 (FL), and 2 cases with 10 movies of diffuse large B-cell lymphoma (DLBCL). We tracked the movement, analyzed the activity of B cells and PD1-positive T cells under reactive conditions, and compared the results to different types of lymphomas. In this study, CD20-positive B cells were examined in the context of CD35 staining. CD35 stains follicular dendritic cells (FDC), an indication for a germinal center, so we primarily analyzed B cells in the germinal center areas and partially in the immediate periphery. We categorized cells by defining track types (Low motion cells, Moving and turning in place, Long distance movement) to describe their movement pattern and action types (Passive cells, Interactive cells, Active cells) to describe their behavior and interactions. In neoplastic tissue, slower cellular dynamics and fewer interactions were observed compared to reactive tissue. This indicates differences in cellular behavior between reactive and neoplastic tissues, with a more dynamic cellular environment observed in reactive tissue.

## Introduction

A strong immune system is essential to protect the body from diseases. There are two main parts of the immune system – the innate and adaptive immune systems. The adaptive immune system includes B and T lymphocytes, and the immune response depends on the interaction of these cells [1–4]. To maintain a healthy immune system, these cells must be mobilized and interact, initiating immunological processes [5]. Therefore, the analysis of such movement and interaction is desirable. After analyzing cells in 2D and 3D, the next step is to observe and analyze the cells in action in 4D.

Observing cells in 2D can provide valuable information about, for example a cell’s size, nucleus, or chromatin distribution as well as snapshots of cell interaction and reciprocal cell position [6]. However, to fully understand a cell’s morphology, it is essential to investigate it in 3D [7,8]. To contextualize this information functionally, it’s necessary to have a 4D representation that analyzes how cells move, interact, and communicate. By imaging cells in 4D, it becomes feasible to analyze their movement, including their directions, speed, interactions, contact times, and morphological alterations [9–11]. While most previous research focused on the 4D exploration of cells in mice, an increasing number of publications have delved into this within human tissue [12]. Examining immune cells within reactive and neoplastic tissue may help to improve special aspects of therapy, especially by testing drugs on moving sections before treading the patient [13].

Under neoplastic conditions, molecular processes and tissue alterations cause modifications and constrictions in the immune system. This can lead to the development of neoplastic cells, changes in the microenvironment, and tissue necrosis. As a result, immunological processes are disrupted, leading to compromised immune system functioning [14,15]. To better understand these conditions within neoplastic tissue, we analyzed the behaviors of B and T lymphocytes in reactive tissue and categorized their movement and action patterns. We then compared these outcomes with different lymphomas to better understand their changed behavior [16].

## Materials and methods

### Cases

The analyzed human tissue included 12 cases with 35 movies of lymphadenitis and 8 cases with 23 movies of hyperplastic tissue from the pharyngeal tonsil (Table 1). There were 4 cases with 16 movies of marginal zone lymphoma (MZL), 3 cases with 19 movies of nodular lymphocyte predominant Hodgkin lymphoma (NLPHL), 3 cases with 6 movies of follicular lymphoma (FL), and 2 cases with 10 movies of diffuse large B-cell lymphoma (DLBCL).

**Table 1:** Number of movies and cells in different cases.

Diagnosis	Cases	Movies	Cells
Lymphadenitis	12	35	3725
Hyperplastic tissue from the pharyngeal tonsil	8	23	5705
MZL	4	16	5237
NLPHL	3	19	6583
FL (grade 1/2)	3	6	1697
DLBCL	2	10	2057

We used antibodies to stain CD20-positive B cells, PD1-positive T cells, and CD35 for FDC.

The study was conducted according to the Declaration of Helsinki and informed consent was obtained from all patients. The ethic committee of Goethe University Hospital agreed on the study (Nr.20-876aV). The data has been anonymized and cannot be traced back. The data was accessed for research purposes from April 24, 2019, to February 17, 2023.

Some cases have already been investigated under other aspects by S. Hartmann (2 cases of LA, 1 case of FL) and P. Wagner (4 cases of hyperplastic tissue from the pharyngeal tonsil) [10,12].

### Tissue preparation and live-cell imaging

The tissue preparation was carried out according to Donnadieu et al. [17]. Following the surgical removal of suspicious lymph nodes, a 2-mm segment of the remaining tissue was embedded in 5% low-gelling-temperature agarose prepared in PBS. Slices measuring 350 μm were created using a vibratome in ice-cold PBS. These slices were then placed onto 0.4-mm organotypic culture inserts within 35-mm Petri dishes containing 1.1 mL of phenol-red-free RPMI 1640.

For antibody staining, slices were stained at 37°C for 15 minutes using specific antibodies.

We used the following antibodies: Alexa Fluor 647-anti-human PD-1 (clone EH12.2H7; BioLegend), FITC-anti-human CD35 (clone E11; BioLegend) and Pacific Blue-anti-human CD20 (clone 2H7; BioLegend). All antibodies were diluted to 10 μg/mL in phenol-red-free RPMI 1640.

Subsequent imaging of the tissue slices was performed using a Leica SP8 confocal microscope furnished with a temperature-controlled chamber at 37°C. The slices were perfused at a rate of 0.8 mL/min with a solution of phenol-red-free RPMI 1640, oxygenated with 95% O2 and 5% CO2.

### Data curation

Next, we analyzed the recorded movies and rated them according to their biological and technical quality. All movies that reached a minimum qualitative standard were included in the study. This means a good contrast, clearly identifiable cells, complete cells and minimal background.

### Cell tracking

Individual cells were tracked using the Spot detection of the software IMARIS Advanced Tracking 9.7 (Bitplane AG, Zurich, Switzerland). A Gaussian filter was applied across all three channels to reduce noise. Additionally, background subtraction was activated. The spot size of CD20-positive B cells was set to 8 μm and 7 μm for PD1-positive T cells. The tracks were filtered for a minimum track duration of 5 minutes.

The design of such pipelines strongly depends on the quality of the underlying image data. In particular, membrane-based staining in dense regions poses challenges, as incomplete staining and overlapping cells can hinder accurate segmentation and tracking. To address these issues, we iteratively reviewed and adjusted the tracking pipeline through manual inspection, optimizing it for the best possible performance under the given conditions.

For further analysis, we exported the following features per track: Track Displacement Length (μm), Track Length (μm), Track Speed Mean (μm/min), Track Straightness, and Track Intensity Mean.

### Temporal cell graphs

In addition to the Imaris-based features per track, we exported the single track positions to calculate so-called temporal cell graphs. Temporal cell graphs are defined here as a set of cell graphs that each represent a point in time within one video. Cell graphs are defined as unit-disc graphs, where nodes represent cells and edges represent potential cell contacts [18]. We used a radius of r = 7 µm to generate the cell graphs. Using the temporal cell graphs, we calculated the number of contacts per track as well as the average contact time. The temporal cell graphs were calculated using a pipeline implemented in Python 3.11 with the NetworkX package, an example can be found in the appendix [19].

### Qualitative investigation and manual annotation

We then manually examined single cells in the individual videos with regard to different behaviours. As part of this analysis, we defined the track and action types described in results. Furthermore, we manually annotated a subset of 1726 tracks from 19 cases of the previously exported dataset according to their corresponding track and action types.

### Data analysis and validation

To automate the annotation of individual track and action types per track, we trained a random forest classifier. For this, we implemented a Python 3.11-based pipeline using the scikit-learn package [20]. We used the following features for training: Track Displacement Length (μm), Track Length (μm), Track Speed Mean (μm/min), Track Straightness, Track Intensity Mean, number of contacts, and mean contact duration (frames). Validation of the random forest classifier resulted in an accuracy of 75,53% for track types and 53,27% for action types in a 20-times stratified cross-validation. However, due to the sometimes fluid delimitation of individual types, we also carried out a manual validation of the classifier. For this purpose, we analyzed 4704 tracks. This showed an accuracy of 88,4% for track types and 78,4% for action types.

## Results

### Definition of cellular subgroups

#### Track types.

**Fig 1:**
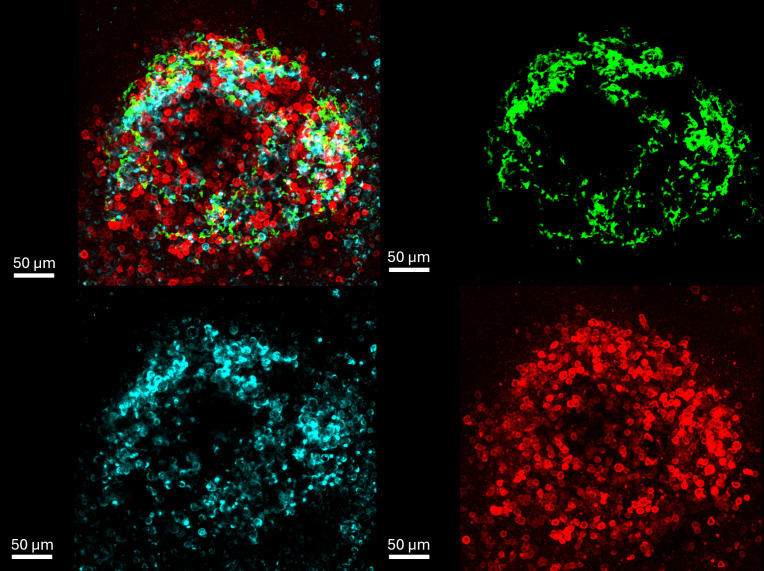
Example of stained tissue. A germinal center from reactive tissue (hyperplastic tissue from the pharyngeal tonsil). FDC network (green), CD20-positive B cells (blue) and PD1-positive T cells (red).

The reactive tissues, including LA and hyperplastic tissue from the pharyngeal tonsil, were analyzed qualitatively to observe the movement patterns and behaviors of the cells. Annotated tracks of CD20-positive B cells and PD1-positive T cells revealed that the cells exhibited variations in movement patterns and speeds. It was observed that cells showed rapid and directional movement, minimal movement, and high activity within the same region. Based on these distinct characteristics, three different track types were defined. The mean velocities were 3,62 µm/min for CD20-positive B cells and 4,91 µm/min for PD1-positive T cells (Fig 1). A more detailed analysis about the velocities can be found in the previous work of Wagner et al. [12] and in S2 Table.

#### Low motion cells (LM).

The cells referred to as “Low motion cells” are characterized by minimal movement and a tendency to remain mostly stationary. During the observation period, these cells were seen to either barely move or not move at all.

**Fig 2:**
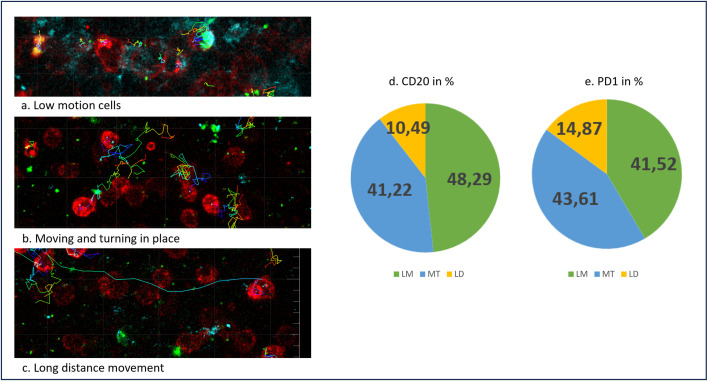
Different track types under reactive conditions. (a.) Track type “Low motion cells” of CD20-positive B cells (blue) and PD1-positive T cells (red). (b.) Track type “Moving and turning in place” of PD1-positive T cells. (c.) Track type “Long distance movement” of one PD1-positive T cell. (d.) Distribution of the different track types for CD20-positive B cells (e.) Distribution of the different track types for PD1-positive T cells.

**Fig 3:**
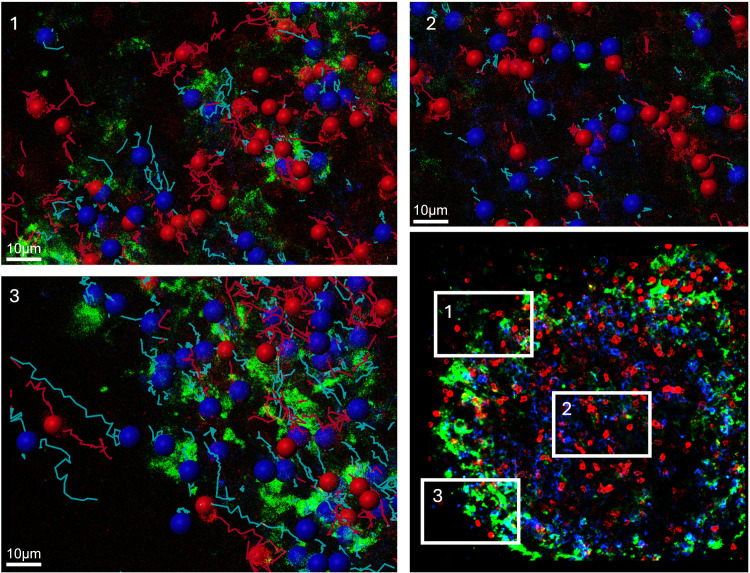
Tracks in different areas. A germinal center from reactive tissue (hyperplastic tissue from the pharyngeal tonsil) with the FDC network (green), CD20-positive B cells (blue) and PD1-positive T cells (red). (1) The tracks of CD20-positive B cells (blue) and PD1-positive T cells (red) at the edge of the germinal center, near the FDC network with mainly MT tracks. (2) The center of the germinal center with mainly LM tracks. (3) The periphery next to the FDC with mainly LD.

Fig 2a gives an example of such cells. As can be seen in the figure, the colored tracks are short, suggesting that the cells barely move. These cells were frequently found in cell clusters, mainly at the center of a germinal center, as shown in Fig 3. and were rarely observed interacting with each other. When movement was observed, it was found to be very slow.

#### Moving and turning in place (MT).

This track type shows localized movements within the same area. The cells move undirected and remain within the same area throughout the movie.

Fig 2b shows the MT track type, which has longer tracks than LM but is still limited to the same area. These cells move a greater distance, but their movements are concentrated nearby. They exhibit back-and-forth or circular motions and frequently interact with other cells, especially MT.

MT are often associated with CD35-positive FDC located at the periphery of a germinal center, as shown in Fig 3.

#### Long distance movement (LD).

In this track type, cells that cover a greater distance and usually display directed movement are observed. They frequently follow a linear trajectory with greater velocity compared to LM and MT.

Fig 2c shows a linearly elongated path, which represents an LD track.

As Fig 3 shows, these cells were mainly detected outside the germinal center, but a small number of cells within the germinal center were also found to cover these greater distances. Compared to the cells located outside the germinal center, those within exhibited a higher tendency to interact with other cells, resulting in more interactions.

#### Action types.

In addition to the different movement parameters, cells interacted differently and changed their morphologies. Three general characteristics were identified, and the cells were divided into three subgroups based on their activities.

#### Active cells (AC).

Mobile cells, characterized by limited interactions, underwent dynamic morphological transformations. Their movement during the movies was correlated with a change in morphology. The cells contracted or expanded, sometimes forming small tails.

**Fig 4:**
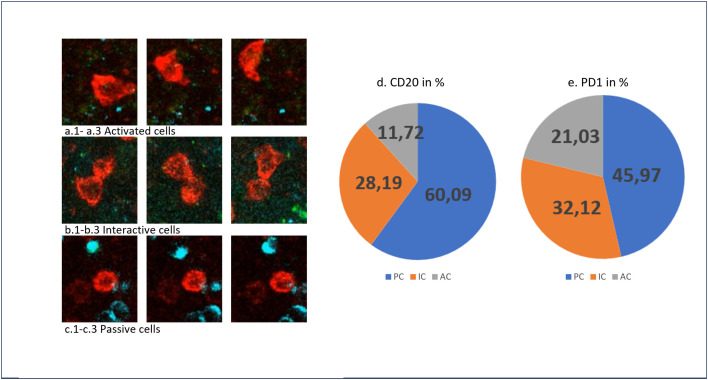
Different action types under reactive conditions. (a.) Action type “Active cells” of one PD1-positive T cell (red). a.1 shows the cell at the beginning of the movie and a.2 and a.3 show its behavior during the movie. (b.) Action type “Interactive cells” of PD1-positive T cells. b.1-b.3 show two T cells interacting with each other during the movie. (c.) Action type “Passive cells” of CD20 positive B cells (blue) and PD1-positive T cells (red). The round cell stays in their round shape during the movie. (d.) Distribution of the different action types for CD20 positive B cells. (e.) Distribution of the different action types for PD1-positive T cells.

These cells were classified as AC and can be seen in Figs 4a, 1-a3 and 5.

**Fig 5:**
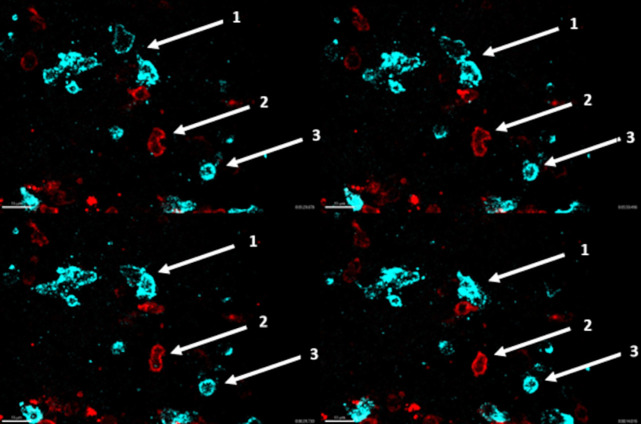
Different frames showing cell behavior. The four snapshots show different time points, captured at 5:28, 5:50, 6:31 and 8:14 minutes. (1) Action type IC of two CD20-positive B cells (blue). (2) Action type AC of one PD1-positive T cell (red). (3) Action type PC of one CD20-positive B cells (blue).

Even in the presence of nearby cells, minimal interactions were observed. These cells were often found to be in an environment with limited proximity to others, engaging in exploratory behavior through wandering.

#### Interactive cells (IC).

The action type “Interactive cells” exhibited cellular behaviors characterized by frequent intercellular interactions. The cells made contact with each other, resulting in changes in their morphology.

Fig 4b.1-b3 and 5 illustrates two cells that actively interact with their surroundings. They engage with neighboring cells, sometimes forming cellular clusters, and have high contact times.

#### Passive cells (PC).

The action type “Passive cells” is characterized by cells with minimal motion and surface alterations.

These cells remained unchanged during the movies, as depicted in Figs 4c.1-c3 and 5. They retained their original morphology throughout the movies, with round cells, as shown in Figs 4c.1-c3 and 5, maintaining their round shape.

They are frequently found in proximity to dendritic cells.

### Comparison of reactive and neoplastic tissue

**Fig 6:**
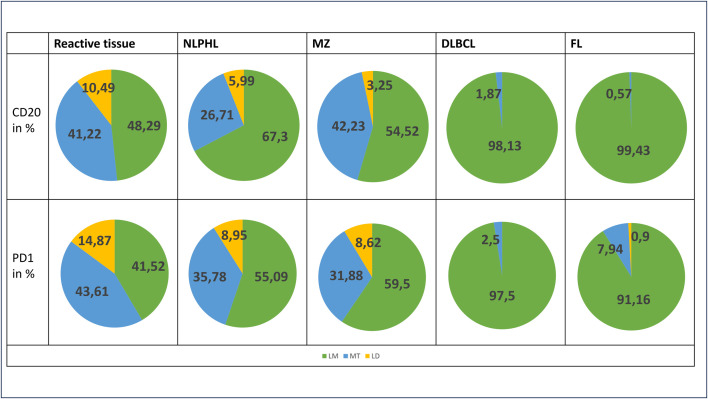
The distribution of track types. The percentage distribution of the track types of the total cells in reactive tissue (LA and hyperplastic tissue from the pharyngeal tonsil), NLPHL, MZ, DLBCL and FL. Green shows LM, blue shows MT and yellow shows LD.

**Fig 7:**
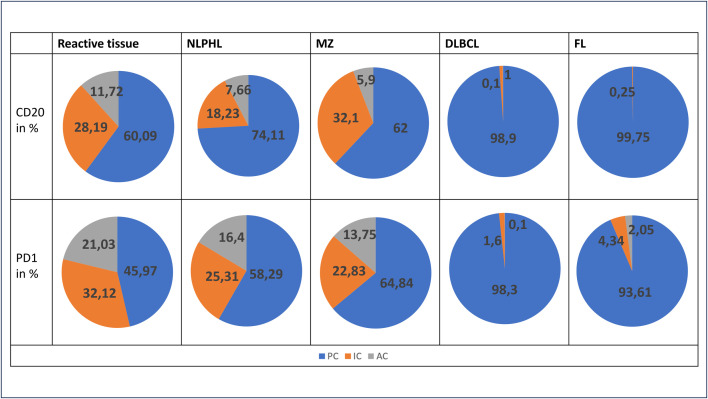
The distribution of action types. The percentage distribution of the action types of the total cells in reactive tissue (LA and hyperplastic tissue from the pharyngeal tonsil), NLPHL, MZ, DLBCL and FL. Blue shows PC, orange shows IC and grey shows AC.

To understand how movement changes under neoplastic conditions, we also extended our analysis to cells within neoplastic tissue. We defined the same types with the same classifier and compared the data we found in the reactive tissue (LA and hyperplastic tissue from the pharyngeal tonsil) with the cells under neoplastic conditions. In general, it shows that cells in neoplastic tissue are slower and exhibit fewer interactions (Figs 6, 7).

#### Characteristics of NLPHL.

Compared to reactive tissue, NLPHL exhibits a higher proportion of PC and a lower occurrence of LD. Additionally, the presence of MT in NLPHL has decreased, albeit still being notable. Consequently, cells within NLPHL demonstrate a reduced overall velocity compared to those in reactive tissue, with diminished movement.

A similar trend in the distribution of action types is evident in NLPHL when compared to reactive tissues, characterized by a predominance of PC. The proportion of IC and AC is lower in NLPHL compared to reactive tissue. However, differences between B and T lymphocytes can be observed. In T lymphocytes, the proportion of IC and AC exceeds that of B lymphocytes, whereas PC dominate with nearly 75%.

#### Characteristics of MZ.

The LM predominate in the MZ, indicating a noticeable increase compared to the reactive tissue. The presence of LD is diminished in the MZ, and T lymphocytes show less MT compared to reactive tissue. Consequently, cells within the MZ exhibit reduced velocity, accompanied by decreased movement.

We observe a heightened presence of PC in comparison to the reactive tissue. The proportion of IC is lower but still present, whereas the presence of AC is less common.

#### Characteristics of DLBCL.

In DLBCL, a distinct contrast is evident in the movement and activity of cells compared to reactive tissue. LM are predominant, with LD no longer observable. MT persist but in a minimal proportion. Overall, there is a notable reduction in cellular movement and activity compared to reactive tissue.

A similar pattern is observed in the action types. PC comprise almost the entire number of cells, whereas the number of IC is reduced, and AC are scarcely found in the tissue.

#### Characteristics of FL.

In FL, cell movement and activity decrease noticeably compared to reactive tissue. A limited number of MT can be identified and a small number of LD for PD1-positive T lymphocytes, but most exhibit LM.

PC represent a substantial portion of the cells, while the proportion of IC is deficient, especially among the B lymphocytes. No AC can be found among these, unlike the T lymphocytes, where a small proportion of AC is still present.

## Discussion

In this study, B and T lymphocytes were observed and analyzed under reactive conditions in the context of CD35. Based on their patterns of movement and behaviors, track types and action types were defined and subsequently annotated using machine learning.

The track types were categorized into three groups: LM, MT, and LD. Action types were classified as PC, IC, and AC. The next step was to apply these categories to various lymphomas and examine the difference between reactive and neoplastic tissue.

Observing the track and action types in a functional context reveals various correlations with the functions of the cells. Different subpopulations during the immune response were described using these track and action types [21].

The movement of lymphocytes probably correlates with their function.

LM cells, characterized by fewer contacts and less displacement, were mainly found in cell clusters, primarily located in the center of germinal centers. These cells were potentially proliferating, although this was not directly investigated in this study. [22]. Moving towards the periphery, MT cells were observed, likely representing B cells searching for an antigen or PD1-positive T cells searching for antigen-presenting B cells [23,24]. A minor proportion of cells performed LD, suggesting a directed movement over longer distances, possibly in search of antigens or as active cells circulating between different zones of the immune response [25].

AC appeared to be actively searching for antigens or other cells for interaction, while IC showed frequent and prolonged contact, suggesting interaction, and signaling exchange. Cells searching for membrane-bounded antigens were likely visualized as IC, whereas cells searching for soluble antigens would be demonstrated as AC. IC could also represent T cells supporting B cells in affinity maturation and class switching, as well as interactions between B and matching T cells during antigen presentation [26–29].

PC cells were characterized by minimal movement and contact, potentially indicating cells undergoing negative selection or resting B cells displaced into the mantle zone. Furthermore, technical errors may occur while examining live tissue and representing it in 4D. Consequently, these cells, for instance, could be decreased.

Reactive tissue generally exhibited more movement and activity compared to neoplastic tissue, with differences observed among different lymphoma types.

NLPHL cases showed shifts in action and track types but still maintained a certain degree of activity, likely due to the presence of reactive B cells in the micro milieu.

Lymphocyte-predominant cells are known to influence their environment, and certain processes are blocked, for instance, in the context of immune evasion. Therefore, the proportions of active track types LD and MT as well as active action types AC and IC are likely to have decreased but are still present [30–32].

The cells in the MZ exhibit a similar behavior to that observed in NLPHL. There is also considerable movement and activity here, although PC and LM predominate. The rare lymphoma displays various histological images, such as neoplastic cells infiltrating and colonizing the remaining GC [33,34].

In DLBCL and FL, predominantly LM and PC cells were observed, probably indicating highly proliferative and mostly neoplastic cells no longer involved in the germinal center reaction [35,36].

For this study, cells were described using spot detection, which does not provide information about cell size. As the cell size is an important aspect of distinguish for example between centrocytes and centroblasts, we were not able to differentiate between different B cell types in this study.

The same limitation applies to cells in neoplastic tissue. For example, in DLBCL, large CD20- positive B cells are likely neoplastic while smaller cells may represent accompanying infiltrates of reactive B cells [36]. Due to technical limitations, we were not able to reliably distinguish between these subsets based on size, which prevented us from differentiating them in our study.

Similarly, in NLPHL, a majority proportion of CD20-positive B cells are reactive B cells. Large cells in the infiltrate, perhaps lymphocyte predominant cells, cannot be fully distinguished from large centroblasts in the germinal centers, as both are positive for the B cell marker we used [29,30].

Overall, malignant diagnoses exhibited less activity and movement compared to reactive tissue. However, this study represents a pilot study with limited cases for various malignant diagnoses, and further research is needed to validate these findings. Nevertheless, specific trends could be identified within the various diagnoses.

## Conclusion

In this study, we observed and analyzed the behavior of B and T lymphocytes in reactive and neoplastic tissue. We examined B and T lymphocytes under reactive conditions in the context of CD35. Using machine learning, we defined track types (LM, MT, LD) and action types (PC, IC, AC) based on their movement patterns. These classifications were applied to different lymphomas to compare reactive and neoplastic tissue.

Reactive tissue generally showed more movement and activity than neoplastic tissue. NLPHL cases exhibited shifts in action and track types but maintained activity due to the presence of reactive B cells. Hodgkin cells influenced their environment, reducing active track and action types. Cells in the MZ showed behavior like NLPHL.

DLBCL and FL predominantly exhibited LM and PC cells, indicative of highly proliferative neoplastic cells. Overall, malignant diagnoses exhibited less activity than reactive tissue.

This study provides important insights into immune-system function and the dynamics of B and T cells in both reactive and neoplastic tissues, offering information on how these cells interact and move in different microenvironments. 4D analysis allows us to investigate the behavior and dynamics of immune cells — processes that are essential to immune system function. Disruptions to these properties can lead to immunodeficiency and functional impairment. This dynamic information cannot be gleaned from static histology or molecular profiling alone. Even detailed molecular analyses and indications of immune activation do not provide insight into whether, or how efficiently, these cells reach their target structures.

By expanding the number of cases and tracked cells, it may be possible to investigate migration, intercellular dynamics, and functional behavior in far greater detail. This could help identifying lymphoma-subtype-specific patterns that may reveal novel therapeutic targets. Once such patterns or cellular subgroups emerge, one feasible next step could be to perform in-vitro drug assays— as previously studied by Yadigaroglu et al.—to evaluate targeted treatments directly on those cells [13]. This could provide valuable information for the design of immunotherapies, particularly in how to modulate immune response to better target tumor cells. Linking these kinetic profiles to molecular data could clarify mechanisms operative in reactive tissue and illuminate how they are altered during malignant transformation. Although our current dataset is anonymized, future studies could include clinical variables such as age, gender, or pre-existing conditions to uncover additional correlations.

## Supporting information

S1 Table Data per track. Exported features, staining, tracktype and actiontype.
(XLSX)

S2 Table Comparison of Cell Velocities with Literature Data. The left column (“Our Work”) presents the mean velocities for reactive T and B cells calculated in our study. The values reported by Wagner et al. and Hartmann et al. represent subsets of the current dataset. All remaining columns refer to independent studies from the literature. It is important to note that these values pertain to different species (e.g., mouse, human), immunological subsets (e.g., follicular helper T cells vs. general T cells), diagnoses (e.g., reactive, neoplastic), tissue types (e.g., lymph node, adenoid), and anatomical regions (e.g., follicular vs. non-localized). As such, direct comparisons are limited. Nevertheless, we present these values as reference points, acknowledging that fully comparable independent studies are not available [10–12,24,29].
(XLSX)

S1 Fig Advanced statistical analysis of reactive B and T Cell Motility. (a) Clustered visualization of individual cell trajectories, aligned to a common origin. (b) Boxplot comparing the mean speeds of reactive B and T cells. (c) Mean squared displacement (MSD, in µm²) and root mean squared displacement (RMSD, in µm) plotted against lag time (in minutes), illustrating diffusive to subdiffusive motion behavior.
(TIF)

S4 Video Example for a movie with stained tissue. Hyperplastic tissue from the pharyngeal tonsil with FDC network (green), CD20-positive B cells (blue) and PD1-positive T cells (red).
(MP4)

S5 Video Example for a temporal cell graph video. The corresponding temporal cell graph video for the S4 Video with CD20-positive B cells (blue) and PD1-positive T cells (red).
(GIF)
