# Supplementary figures and images for: Behavioral investigation of reactive and neoplastic lymphocytes in human lymph nodes in 4D
Source: PLoS One. 2025 Sep 4;20(9):e0331439. doi: 10.1371/journal.pone.0331439 (PMC12410774; doi:10.1371/journal.pone.0331439)

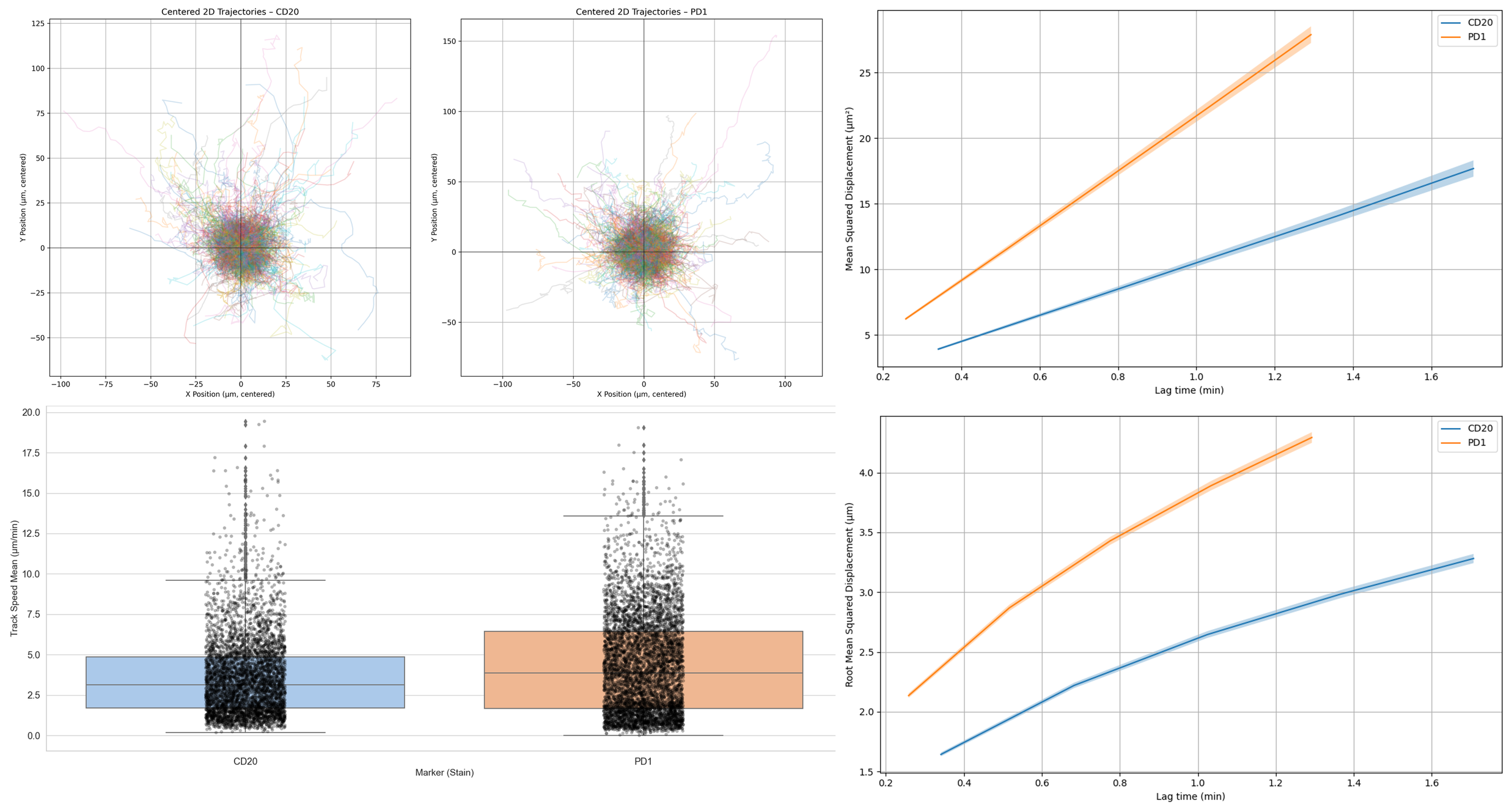

Supplement: S1 Fig — (a) Clustered visualization of individual cell trajectories, aligned to a common origin. (b) Boxplot comparing the mean speeds of reactive B and T cells. (c) Mean squared displacement (MSD, in µm²) and root mean squared displacement (RMSD, in µm) plotted against lag time (in minutes), illustrating diffusive to subdiffusive motion behavior. (TIF) [file pone.0331439.s003.tif]

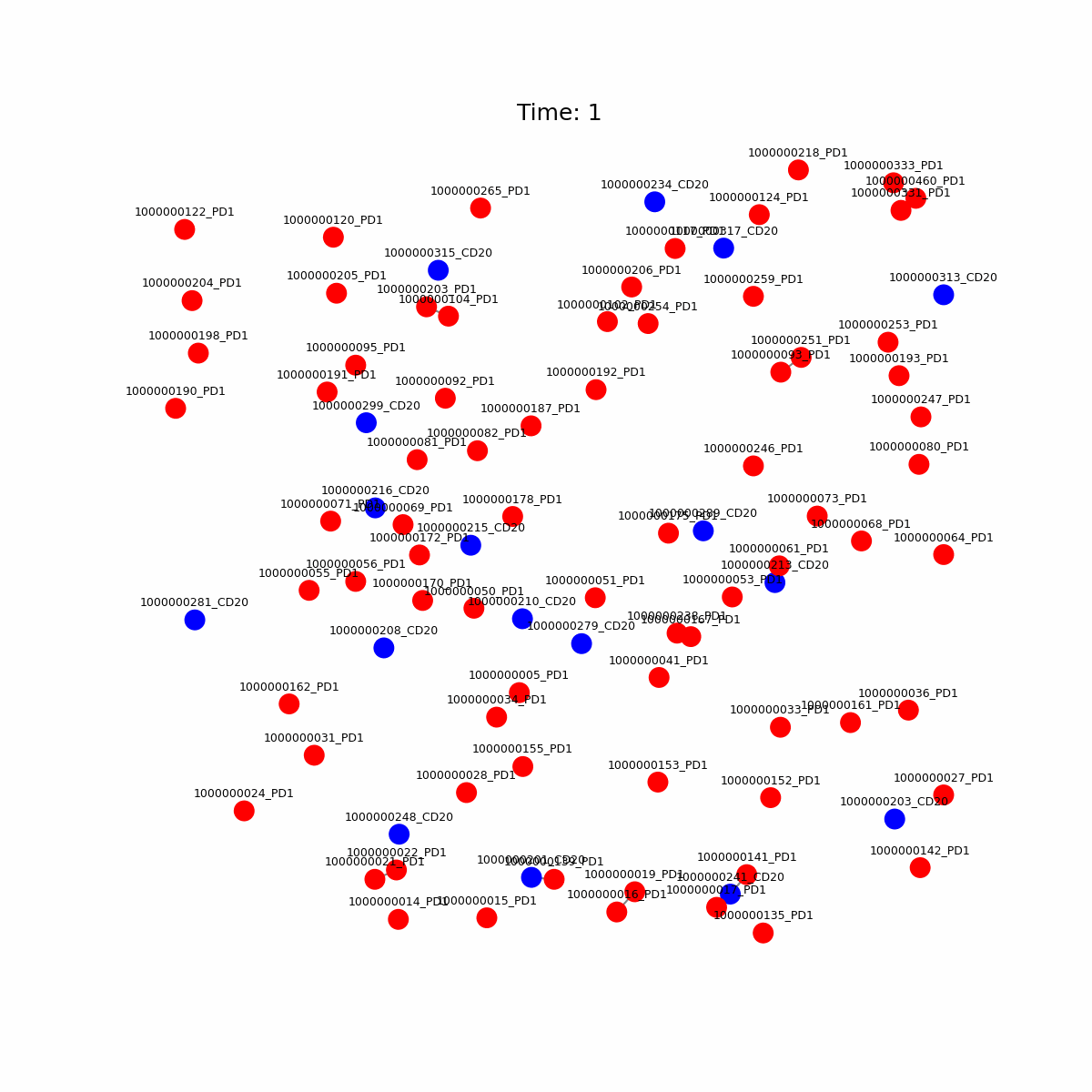

Supplement: S5 Video — The corresponding temporal cell graph video for the S4 Video with CD20-positive B cells (blue) and PD1-positive T cells (red). (GIF) [file pone.0331439.s005.gif]
